# Persistent Postural-Perceptual Dizziness: Precipitating Conditions, Co-morbidities and Treatment With Cognitive Behavioral Therapy

**DOI:** 10.3389/fneur.2021.795516

**Published:** 2021-12-24

**Authors:** John Waterston, Luke Chen, Kate Mahony, Jamila Gencarelli, Geoff Stuart

**Affiliations:** ^1^Department of Neuroscience, Central Clinical School, Monash University, Melbourne, VIC, Australia; ^2^Oto-Neurology Department, Alfred Hospital, Melbourne, VIC, Australia; ^3^Private Practice, Lyttleton Street Medical Clinic, Castlemaine, VIC, Australia; ^4^Department of Cognitive Science, Macquarie University, Sydney, NSW, Australia; ^5^School of Psychological Sciences, University of Melbourne, Parkville, VIC, Australia

**Keywords:** persistent postural perceptual dizziness (PPPD), cognitive behavioral therapy (CBT), dizziness, treatment, investigations

## Abstract

Persistent postural perceptual dizziness (PPPD) is a common chronic vestibular disorder characterized by persistent vestibular symptoms, including postural instability and non-spinning vertigo, which is aggravated by motion, upright posture and moving or complex visual stimuli. In our review of 198 cases seen over a 5 year period, we have confirmed a number of common precipitating conditions for PPPD, including anxiety disorders and vestibular migraine. Vestibular abnormalities, including a unilateral loss of vestibular hypofunction and isolated otolith abnormalities, were found on investigation in just under half the cases. The use of cognitive behavioral therapy (CBT) as a treatment for PPPD resulted in impressive reductions in anxiety and measures of dizziness over follow up periods of up to 6 months.

## Introduction

PPPD is a form of chronic functional neurological disorder which displays many features of a sensory misperception or mismatch syndrome. The condition was added to the Barany Society Classification of Vestibular disorders in 2017 (1), but many of the clinical features have almost certainly been described under different diagnostic entities since the 19^th^ century (1). Previously used diagnostic terms such as agoraphobia, space and motion discomfort, psychogenic dizziness, phobic postural vertigo, visual vertigo and chronic subjective dizziness share many of their clinical features with PPPD.

The diagnosis of PPPD is based on the presence of one or more symptoms of dizziness, unsteadiness, or non-spinning vertigo that has been present on most days for 3 months or more, with exacerbation by upright posture, active or passive movement, and exposure to moving or complex visual stimuli. Dizziness refers to a sensation of disturbed or impaired spatial orientation (2), rather than its more common use as a non-specific umbrella term that includes many different sensations such as faintness and vertigo. The diagnostic criteria also specify that PPPD is usually triggered by another vestibular disorder, medical illness or psychological distress, and also causes significant distress or functional impairment.

In this paper, we describe the breakdown of co-morbidities, investigation results and response to treatment with CBT.

## Materials and Methods

A retrospective review of 198 cases personally assessed and examined by 2 clinicians (JW and LC) over a period of 5 years was examined to document precipitating conditions, co-morbidities and investigation results. All cases fulfilled the Barany Diagnostic Criteria for PPPD (1). A number of neuro-otological investigations were performed including audiometry, caloric testing, video head impulse testing, and rotational chair testing. Otolith function was also assessed with tests of subjective visual horizontal (static bias) and cervical and ocular vestibular evoked myogenic potentials (cVEMP and oVEMP). Tests of central pathways were also conducted (smooth pursuit, vestibulo-ocular reflex suppression and saccadic analysis).

Patients were counseled regarding the diagnosis, precipitating conditions, and nature of the abnormal sensory processing which frequently resulted in an abnormal dependence on visual over vestibular cues for postural stability. The interplay of psychological factors, particularly anxiety, was also discussed, while also emphasizing the physiological basis for many of the symptoms.

A subgroup of 150 cases was assessed and treated with a specialized form of cognitive behavioral therapy (CBT) by a neuropsychologist (KM) and the results of this treatment are reported. Patients were offered up to 6 sessions of CBT (mean 4.8).

When counseling patients about the use of CBT in the treatment of PPPD, it is important to explain that the symptoms are part of a maladaptive process of abnormal sensory processing and postural control, in response to stress or another medical or vestibular disorder, which has become habitual and that these unhelpful strategies can be changed over time. The relationship that patients have with PPPD symptoms is often antagonistic or fearful and may involve self-limiting beliefs regarding recovery. An important aspect of the treatment is to encourage patients to see the symptoms as non-threatening.

Patients were asked to list specific avoidance behaviors because of their PPPD symptoms which helped to guide the behavioral component of the work. Such factors as fear of falling, social embarrassment, avoidance, and safety behaviors were addressed through explanation of high-risk postural control and other responses that had become maladaptive and which were contributing to the vicious cycle of symptoms. Gait was observed and, if possible, video-recorded with the patient's permission and compared to footage of their gait with low-risk alterations. In-session behavioral experiments and activities were used to demonstrate and explain features such as distractibility and reversibility. Mindfulness or relaxation techniques were used as tools for reducing symptoms, particularly at times when subjects were undertaking graded exposure tasks.

Dizziness symptoms (DSI) (3), dizziness handicap (DHI) (4), avoidance and safety behaviors (3) and anxiety (DASS-21) (5) scores were calculated before and immediately after treatment, as well as some preliminary data for 6 month follow up.

## Results

Of the 198 cases originally selected to examine precipitating conditions, co-morbidities and investigations results, 136 (69%) were female. The age range was 16 to 87 years, with the average age being 47.6 years. Of the subgroup managed with CBT, the mean age was 46. The median symptom duration was 1.5 years, ranging from 3 months to 35 years with an average of 3.5 years, placing this group in the middle range of other published studies regarding illness duration.

**Figure 1:**
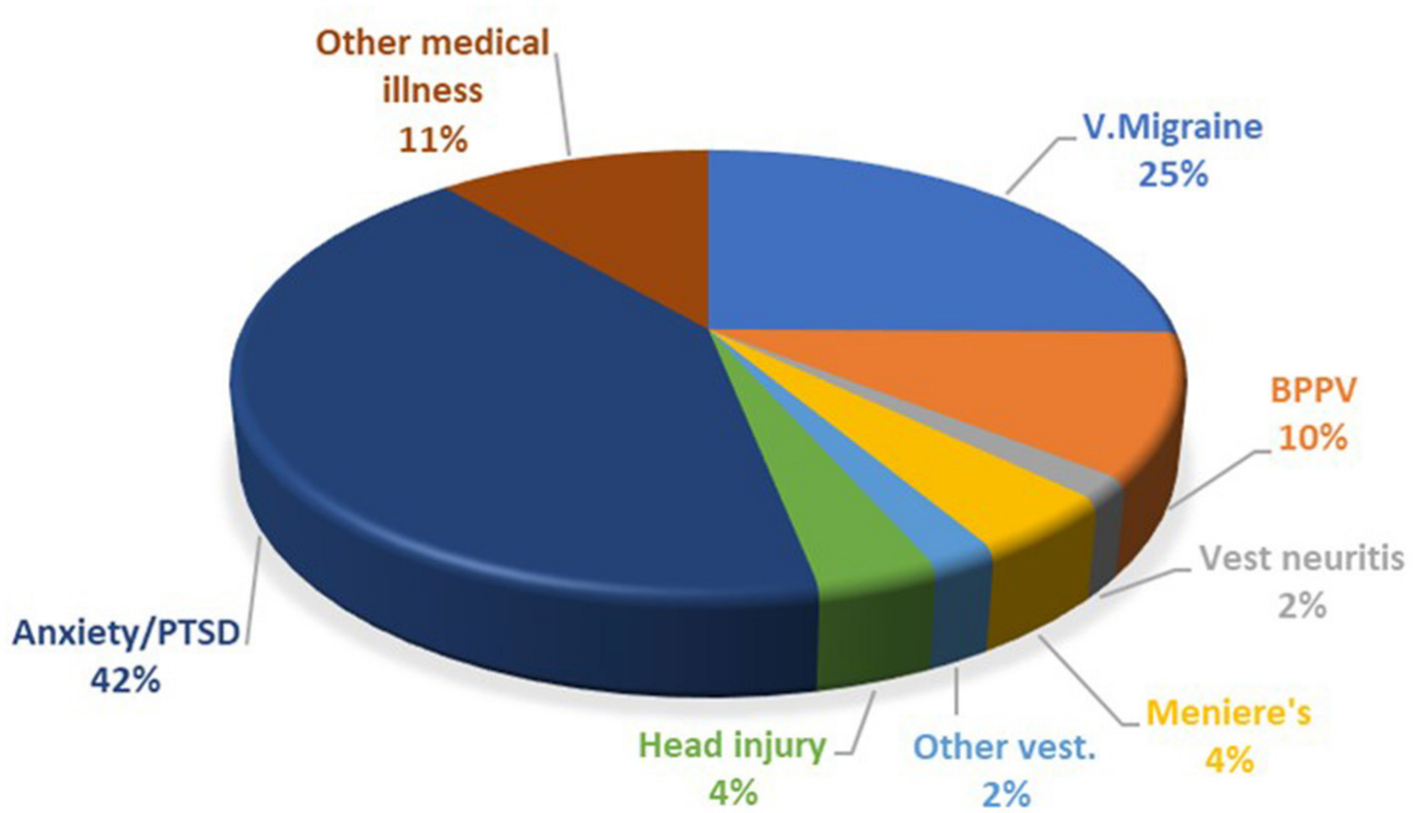
Breakdown of PPPD co-morbidities. PTSD, post-traumatic stress disorder; V. Migraine, vestibular migraine; BPPV, benign paroxysmal positional vertigo.

The breakdown of the precipitating conditions is shown in Figure 1. Of note, the most common associated vestibular condition was vestibular migraine, being present in 25% of cases. Psychological distress in the form of anxiety and post-traumatic stress disorder (PTSD) was present in 42% of cases. In many other cases anxiety also developed as a complication of PPPD.

**Figure 2:**
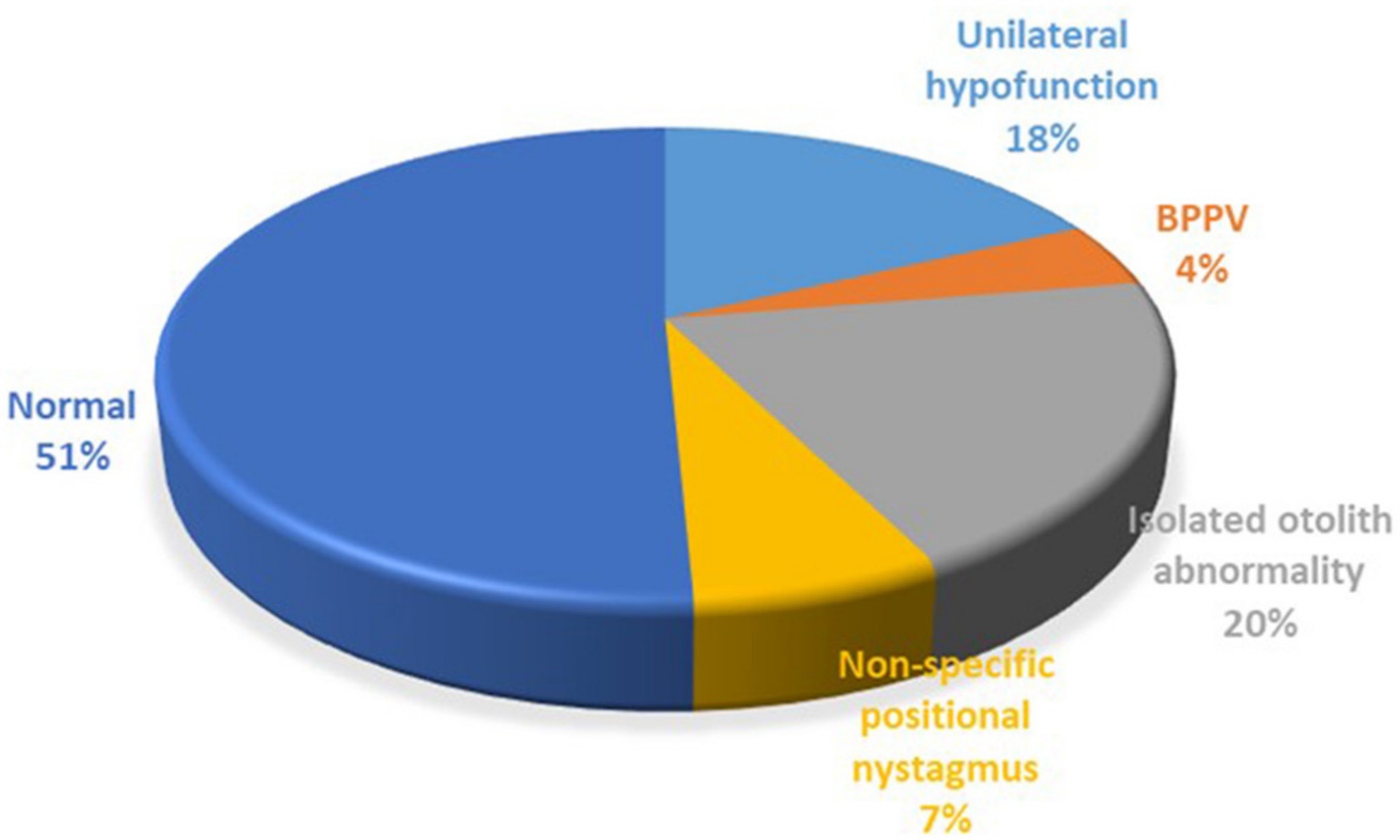
Investigation results. BPPV, benign paroxysmal positional vertigo.

Neuro-otological investigations were performed in 126 patients (Figure 2). The majority of patients had normal results (51%), though there were also many patients with vestibular abnormalities, including a significant number with isolated unilateral otolith abnormalities based on static bias, cVEMP and oVEMP results. Central pathway abnormalities, mostly mild, were found in 6 patients only, and these results did not change the underlying diagnosis of the precipitating condition.

Of the 186 patients who were referred for CBT, 8 patients dropped out after the first CBT session and 28 patients did not complete the treatment program. Many in the latter group had other significant psychological or psychiatric comorbidities. Of the study group, 100 patients had at least tried medication including selective serotonin reuptake inhibitors, serotonin and norepinephrine reuptake inhibitors, tricyclic antidepressants and/or vestibular rehabilitation.

Table 1 shows analysis of the pre- to post- treatment reduction in PPPD symptoms, disability, avoidance and safety behaviors, and anxiety. The pre-treatment means place the group in the moderate range of handicap with respect to the Dizziness Handicap Inventory and the moderate range of anxiety on the DASS-21.

**Table 1:** Means, SDs, Cohen's *d* effect sizes and paired t-tests on outcome measures at pre- and post-treatment (*n* = 150).

**Outcome**	**Mean**	**SD**	**Effect**	**95% confidence**	***t*** **-value**
**measure**			**Size**	**interval**	
**DSI**
Pre-treatment	29.94	12.98	1.29	27.84–32.03	−18.02***
Post-treatment	15.23	9.63		13.68–16.79	
**DHI**
Pre-treatment	50.03	20.80	1.33	46.65–53.40	−19.32***
Post-treatment	23.92	18.82		20.86–26.98	
**ASB**
Pre-treatment	20.57	12.09	1.5	18.64–22.54	−15.64***
Post-treatment	7.72	7.41		6.52–8.92	
**Anxiety**
Pre-treatment	12.76	9.15	0.87	11.26–14.27	−10.72***
Post-treatment	6.1	6.09		5.08–7.09	

*** *p = 0.0005. DSI, Dizziness Symptom Inventory; DHI, Dizziness Handicap Inventory; ASB, Avoidance and Safety Behaviors; Anxiety, DASS-21 Anxiety scale*.

Large effect sizes and significant differences were seen across all four measures, comparable to those reported by other studies using brief CBT (3). The large effect size for anxiety is an improvement on other studies that have used fewer treatment sessions (3).

Given the single group pre-post design, we also used individual based change (IBC) statistics to identify specific individuals who showed statistically reliable change, as suggested by Estrada et al. (6). If the pattern of individual changes is normally distributed, the proportion of individuals that show reliable change is a direct function of the shift of the normal distribution from a mean of no change. However, this is not the case when data are not normally distributed (e.g., skewed) or if there are outliers. In addition, the proportion of individuals who show statistically reliable individual change, even from a normal distribution, is more clinically interpretable than Cohen's *d* or *t*-values that assume homogeneity of change. The reliable change index (RCI) of Jacobson and Traux (7) does not assume equality of pre- and post-test variances and was used for that reason. Statistically reliable change is different to clinically meaningful change, however it is interesting to estimate the proportion of patients who became reliably better (not excluding placebo effects), as well as those who became reliably worse.

**Figure 3:**
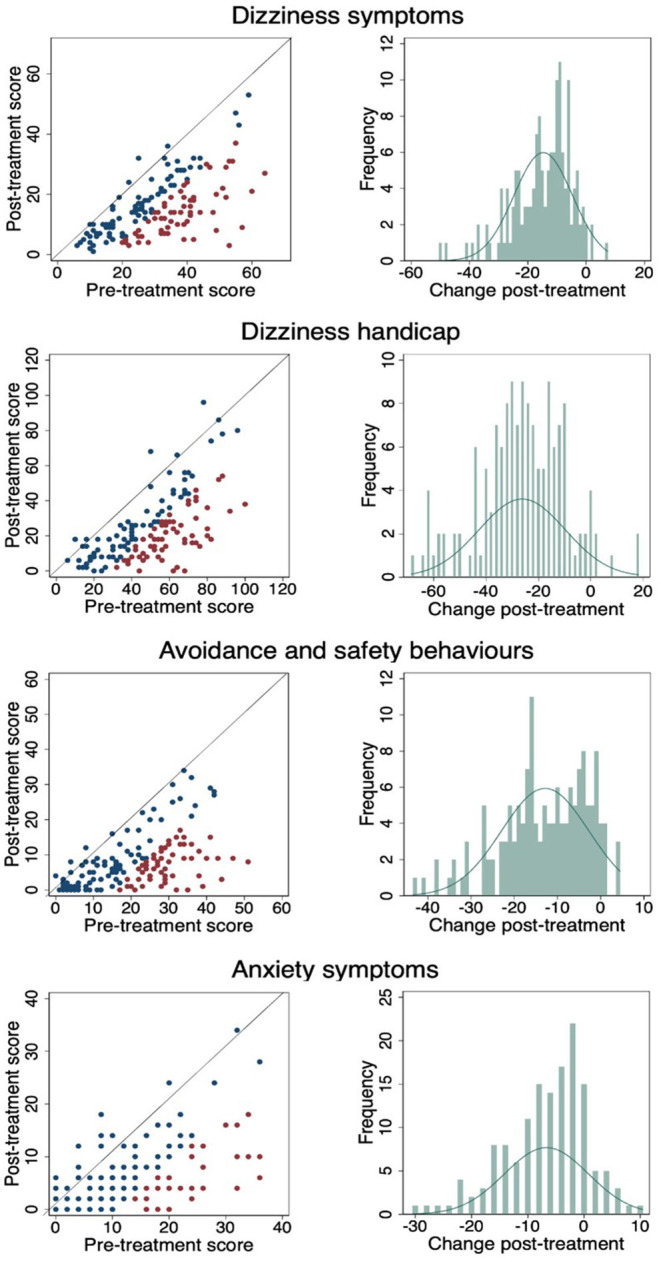
Pre- and post-CBT results and change frequencies for each outcome measure. (Red dots are statistically reliable change, blue dots are non-reliable change and diagonal line represents no change).

Figure 3 shows a scatterplot of the change in individual pre- and post-CBT scores in which the red dots are statistically reliable change, the blue dots are statistically non-reliable change, and the diagonal line represents no change. The percentage of pre-post CBT reliable changes are: DSI- 42%, DHI 45.33%, ASB 40.67% and Anxiety 27.33%. Change frequencies (pre-CBT minus post-CBT) are presented for each outcome measure in the accompanying histogram. As demonstrated, across each of the four outcome measures almost everyone improved to a greater or lesser degree.

Preliminary six-month follow-up data from 49 cases demonstrates significant additional average improvement between post-treatment and 6-month follow-up (Table 2). Given the low sample and effect size/s, it is noted that none of these changes were statistically reliable.

**Table 2:** Means, SDs, Cohen's *d* effect sizes and paired *t*-tests on outcome measures at post-treatment and 6 month follow-up (*n* = 49).

**Outcome**	**Mean**	**SD**	**Effect**	**95% confidence**	***t*** **-value**
**measure**			**Size**	**interval**	
**DSI**
Post-treatment	14.16	8.87	0.52	11.62–16.71	−2.86**
Follow up	9.86	7.56		7.68–12.03	
**DHI**
Post-treatment	23.47	18.38	0.54	18.19–28.75	−2.96**
Follow up	14.78	13.69		10.84–18.71	
**ASB**
Post-treatment	7.89	6.38	0.37	5.71–10.08	−1.84*
Follow up	5.31	7.61		3.47–7.14	
**Anxiety**
Post-treatment	6.36	5.74	0.69	4.72–8.02	−3.26**
Follow up	2.98	3.96		1.84–4.12	

* *p < 0.05*

** *p < 0.005. DSI, Dizziness Symptom Inventory; DHI, Dizziness Handicap Inventory; ASB, Avoidance and Safety Behaviors; Anxiety, DASS-21 Anxiety scale*.

## Discussion

Although the majority of cases of PPPD develop as a result of previously noted precipitating conditions, occasional cases appeared to begin without any obvious trigger. Caution should be exercised in cases of slowly progressive onset without a clear onset precipitating condition as other neurological conditions, particularly neurodegenerative disorders, may be confused. False sensations of “rocking”, “swaying” or “bouncing” and visual motion sensitivity can also be seen in the condition of mal de debarquement which is typically triggered by prolonged vehicular motion. However, in this condition, the symptoms characteristically reduce during passive motion, in contrast to the passive motion aggravation that occurs in PPPD (8).

The most common associated vestibular disorder was vestibular migraine, an observation which may be at least partially explained by the presence of abnormal sensory processing and visual motion sensitivity in both vestibular migraine (9, 10) and PPPD (11).

There was also a high incidence of anxiety and PTSD in our study. Psychological comorbidities, including anxiety, depression and obsessive personality traits have been described as cardinal features in the setting of the related condition of phobic postural vertigo, and it has been postulated that there is an anxiety-related conscious awareness of discrepancies between anticipated and actual movements that occurs transiently in the course of normal voluntary motion, a form of efferent-afferent mismatch (12). The association of anxiety may also be explained by physiological factors. In a study of patients with anxiety disorders, an association between higher space and motion discomfort and increased reliance on somatosensory information for postural control was documented (13). Likewise, in studies of visual vertigo, an abnormally large reliance on visual cues for spatial disorientation was found (14, 15). A review of neuroimaging studies has also supported the hypothesis of visual dependence and anxiety-related mechanisms on locomotor control and spatial orientation (16).

It is also interesting to note that another condition, visual snow, consisting of persistent visual disturbance described as constant flickering dots or static, has also been postulated to be a syndrome of impaired sensory processing which may share a similar pathophysiology with PPPD and other conditions such as migraine and tinnitus (17, 18). PPPD and visual snow also share high rates of psychiatric dysfunction including anxiety (1, 19). A high incidence of balance disorders has been reported in a large study of visual snow (20), and dizziness has also been noted in up to 30% of cases (O.White personal communication).

Vestibular investigations are not needed to make the diagnosis of PPPD but are useful in some cases to exclude or diagnose other underlying vestibular disorders. The cross-section of investigation results is not surprising. Many patients with primary anxiety or vestibular migraine would be expected to have normal results. Peripheral vestibular abnormalities are also well described associations. One surprising finding was the significant finding of cases with isolated otolith abnormalities. While some caution needs to be exercised in interpreting these abnormalities, it is interesting to note the association, given the importance of otolith function in postural control.

Prior to presentation and diagnosis, many patients had already undertaken trials of vestibular rehabilitation therapy with some but limited benefit. While many had noted a modest initial symptom improvement with this treatment, other forms of therapy were usually necessary to produce substantial symptomatic improvement. Nevertheless, patients were encouraged to continue their vestibular exercise therapy while undergoing other treatments to improve balance and mobility, and to reduce motion-induced dizziness. Antidepressant medical therapy including selective serotonin reuptake inhibitors, serotonin and norepinephrine reuptake inhibitors and tricyclic antidepressants were considered along with CBT. The choice of initial treatment between CBT and medical therapy was made after discussion with the patient and was often influenced by other factors including geographical access and availability. Many patients required a combination of medication and CBT.

The large effect size for reduction in anxiety symptoms after CBT is greater than that seen in studies using fewer sessions of CBT or monotherapies (3) and similar to others with combined treatments of medication/CBT or vestibular rehabilitation (21). The smaller 6-month follow up sample revealed further improvements across all parameters. Other studies have demonstrated a stabilization of anxiety, but not further improvement (3). One likely hypothesis is that continued use of medication and/or vestibular rehabilitation and/or CBT tools over a 6 month period builds on gains during treatment with consolidation of a more positive and adaptive cycle. Alternatively, it may be that offering more CBT sessions is better at addressing anxiety in the longer term.

The overall aim of CBT was to encourage subjects to re-focus on meaningful and productive activities. This frequently meant acceptance of a certain degree of dizziness and anxiety. In our experience, and in published studies, the most common outcome is reduction of PPPD symptoms rather than elimination. The overall goal was therefore explained as a functional recovery in the face of persistent but diminishing symptoms and impairment. Some spectacular results were often achieved, even in patients who had been experiencing symptoms for many years.

One potential weakness of this study was the lack of a control group. However, we concluded that the improvements noted were not solely related to placebo effects. In the previously mentioned study of CBT (3), there was a control group, but the placebo effect was much smaller than the effect seen in our study. Many of our patients were also resistant to previous treatments, making it less likely that the improvements were related to placebo alone.

The diagnosis of PPPD was generally well accepted by the great majority of patients, after explanation of the possible physiological basis for the development of symptoms and the contribution of psychological factors. In contrast, previously used diagnostic labels, such as psychogenic dizziness and phobic postural vertigo, are often more difficult for patients to accept as the symptoms are often perceived to have a primary psychological basis. In our experience, the acceptance and understanding of the diagnosis offers the greatest opportunity for recovery with appropriate therapy, particularly with the use of CBT. Recently, we have also found that CBT was able to be performed successfully using video telehealth, after this mode of treatment became necessary during the Covid-19 pandemic.

## Data Availability Statement

The original contributions presented in the study are included in the article/supplementary material, further inquiries can be directed to the corresponding author/s.

## Author Contributions

JW and LC diagnosed, investigated and treated the patient group. JW wrote the initial draft of the paper. JG performed the oto-neurology investigations and analysed these results. KM performed the cognitive behavioral therapy and undertook the initial analysis of the results of this treatment. GS undertook further analysis of the results and provided the statistical models for this work. All authors contributed to the article and approved the submitted version.

## Funding

This article is being funded by the Visual Snow Initiative.

## Conflict of Interest

The authors declare that the research was conducted in the absence of any commercial or financial relationships that could be construed as a potential conflict of interest.

## Publisher's Note

All claims expressed in this article are solely those of the authors and do not necessarily represent those of their affiliated organizations, or those of the publisher, the editors and the reviewers. Any product that may be evaluated in this article, or claim that may be made by its manufacturer, is not guaranteed or endorsed by the publisher.
